# Sino-Nasal 5 Questionnaire is Associated with Poor Asthma Control in Children with Asthma

**DOI:** 10.3390/children4070054

**Published:** 2017-06-28

**Authors:** Sasikumar Kilaikode, Prateek J. Shukla, Gurpreet Phull, James H. Jackson, Dominique C. Prue, Claudia Martinez, Krista Scheffey, Dinesh K. Pillai

**Affiliations:** 1 Division of Pulmonary and Sleep Medicine, Children’s National Health System, Washington, DC 20010, USA; scheruve@childrensnational.org (S.K.); pshukla01@hotmail.com (P.J.S.); JHJackso@childrensnational.org (J.H.J.); dprue@childrensnational.org (D.C.P.); claudiamartinez0926@gmail.com (C.M.); scheffeyk@gmail.com (K.S.); 2 Division of Pulmonary and Sleep Medicine, MedStar Georgetown University Hospital, Washington, DC 20007, USA; Gurpreet.Phull@gunet.georgetown.edu

**Keywords:** asthma, rhinosinusitis, screening, Sino-Nasal 5 score

## Abstract

Up to 80% of asthmatic children may experience upper airway symptoms which are often perceived as coming from the lower airways. Currently, there are no validated questionnaires to assess upper airway contribution to pediatric asthma symptoms. The Sino-Nasal 5 (SN-5) questionnaire was previously validated for identifying radiographic confirmed sinus disease in children. In this study, we hypothesize that significant SN-5 scores (≥3.5) are associated with abnormal National Asthma Education and Prevention Program (NAEPP) based asthma impairment and control in asthmatic children. Retrospective data collected on children with asthma referred for pulmonary evaluation included age, gender, ethnicity, NAEPP asthma severity, asthma control (Test for Respiratory and Asthma Control in Kids (TRACK) < 5 years, Asthma Control Test (ACT) 5 years) and pulmonary function testing. Associations between SN-5 scores and asthma impairment and control were identified. Seventy-six children were evaluated; 38% were female with a mean age of 6.9 years. Significant SN-5 scores were associated with decreased control of daytime symptoms (odds ratio (OR): 0.16 (95% confidence interval (CI): 0.06–0.44)), night time awakenings (0.09 (0.03–0.29)), activity interference (0.2 (0.06–0.68)), NAEPP defined asthma control (0.32 (0.12–0.85)) and poor asthma control based on TRACK (*p* < 0.001) and ACT (*p* < 0.001). This suggests upper airways may play a larger role in perceived lower airway symptoms, and SN-5 may be beneficial in assessing the contribution of upper airway conditions on asthma control.

## 1. Introduction

Chronic rhinosinusitis (CRS) and asthma are two comorbid conditions that lead to pathologic clinical disease affecting the respiratory tract. Up to 80% of children with asthma may experience upper airway symptoms, including rhinitis, which may be perceived as coming from the lower airway [1]. As such, medical management and control of perceived asthma symptoms by treating only the lower airway may be suboptimal. Rhinosinusitis affects the upper airways, specifically the nose, nasal passages, and paranasal sinuses (frontal, sphenoid, ethmoid and maxillary). In contrast, asthma affects the intra-thoracic lower airways, which include the bronchi and bronchioles. Ultimately, the airway proves to be one contiguous structure, functionally working as a continuum between extra and intra-thoracic respiratory structures. Localization of symptoms and their subsequent treatment is often difficult in children with asthma and upper airway co-morbid conditions.

As the most common chronic lung disease of childhood, asthma accounts for major healthcare utilization and economic burden both in the United States and worldwide [2]. Affecting more than 6.6 million children in the United States, children with asthma incur, on average, a 2.8-fold greater yearly healthcare expenditure when compared to those without asthma [3]. The National Asthma Education and Prevention Program (NAEPP) published guidelines for the diagnosis, classification and management of asthma in children are currently in use internationally [4]. The guidelines stratify asthma into groupings on the basis of impairment and risk. Impairment includes daytime symptoms, nighttime symptoms, activity limitation and lung function. Risk is primarily based on systemic corticosteroid administration for asthma exacerbations. Based on findings, asthma is categorized as intermittent, mild, moderate and severe persistent. Monitoring of asthma control can be further classified as well controlled, not well controlled and very poorly controlled based on similar assessments. Despite advances in treatment, awareness and education, asthma management continues to be a challenge for health care providers.

An important step in asthma management is identifying and treating comorbidities that can lead to difficulty obtaining symptomatic control. Comorbidities include infection, obesity, gastroesophageal reflux, allergies and obstructive sleep apnea, all of which can cause upper airway symptoms. CRS is one of the main comorbid conditions affecting severity and control of asthma in children. It has been shown that successful treatment of CRS may positively affect lower airway disease [5]. NAEPP guidelines don’t provide a standardized method to adequately address the impact of upper airway symptoms on asthma control and management.

Quality of life (QOL) questionnaires have gained popularity in medical practice as a meaningful way to ascertain and quantify the impact of health care when a cure is not possible. The Sino-Nasal 5 (SN-5) quality of life questionnaire is a validated QOL tool for managing CRS in children up to 12 years old, and has been studied in teenagers as well [6,7,8,9]. Similar to the Sino-Nasal Outcome Test in adults, SN-5 helps to define symptoms, measure rhinosinusitis health status and appraise quality of life, which is important for the complete assessment of treatment [6]. In this study, we aim to assess the utility of this screening tool in all pediatric patients with asthma seen in the outpatient setting. SN-5 consists of questions in five domains including symptoms of infection, nasal obstruction, allergy, emotional distress and activity limitation. SN-5 has been used to correlate CRS symptoms with sinus disease confirmed by computerized tomography (CT) scan in children undergoing functional endoscopic sinus surgery [10,11]. As symptoms of CRS can mimic asthma, the use of this questionnaire can help identify symptoms consistent with CRS in patients being evaluated for asthma symptoms. To date, there have been no studies assessing the correlation of SN-5 scores with asthma symptoms and control in children.

The purpose of this study is to evaluate SN-5 scores with asthma control as defined by NAEPP guidelines in children including the Asthma Control Test (ACT) and Test for Respiratory and Asthma Control in Kids (TRACK) questionnaires; validated tools for assessing symptom control in children with asthma [12,13]. Poorly controlled asthma with significant SN-5 scores might lead to further identification and better management of the upper airway, ultimately leading to improved control of asthma symptoms and decreased need for asthma therapies. In this study, we explore associations between the Sino-Nasal 5 quality of life questionnaire and asthma impairment and control in children. We hypothesize that children with abnormal SN-5 scores are more likely to have uncontrolled asthma. 

## 2. Materials and Methods

We performed a retrospective chart review of children 1 to 19 years of age referred to a tertiary pediatric pulmonology clinic for persistent asthma. Patients were referred for expert opinion, evaluation and treatment. Data was collected through the clinical and financial data system (Trendstar, McKesson, San Francisco, CA, USA), physician billing system (PracticePoint Manager, McKesson, San Francisco, CA, USA), and electronic medical record (Cerner Millennium, Cerner Corp., Kansas City, MO, USA). Collected data included age, gender, body mass index (BMI) percentile, daytime and night time symptoms, associated medical or surgical problems, medications at time of initial consultation, allergies, family and social histories. Other data collected included results of physical examination and pulmonary function testing. NAEPP guidelines were used for diagnosis, classification and assessment of asthma control. All subjects had already been initiated on inhaled steroids prior to their evaluation in the pulmonary clinic. Further assessment of asthma control utilized previously-validated standardized questionnaires, including either ACT for children greater than 5 years of age, or TRACK for children less than 5 years of age. The impact of CRS was evaluated through completion of the SN-5 questionnaire. The questionnaire was administered by a research assistant blinded to co-morbid conditions. Additionally, the clinician was blinded to SN-5 score results at the time of clinic visit. Subjects were identified as having physician-diagnosed allergic rhinitis if either previously diagnosed clinically or having recurrent symptoms at the time of visit (sneezing, rhinorrhea, nasal obstruction, with or without facial itching). 

Children with diagnoses related to cardiac, immunologic, and rheumatologic disorders were excluded, as these comorbid conditions may affect medical management and other primary outcomes. Comorbidities were identified based on International Classification of Diseases, 9^th^ Revision codes [14]. This included congenital heart defects (codes 745 and 746), cardiomyopathy (425), pericarditis (420, 423), myocarditis (422, 429.0), endocarditis (421), valvular heart disease (424), lupus (710.0), rheumatoid arthritis (714.xx), Goodpasture’s (446.21), granulomatosis with polyangiitis (446.4), vasculitis (447.6), and primary immunodeficiency (279).

Primary analysis evaluated for associations between SN-5 scores and each level of NAEPP asthma impairment: daytime symptoms, night time awakenings, normal activity interference and spirometry. Based on previous validated studies, a significant SN-5 score was defined as ≥3.5 out of a maximum of 7 [11]. These scores have been associated with radiologically confirmed sinus disease [10]. Pulmonary function tests and fractional exhaled nitric oxide (FeNO) measurements were performed in children greater than or equal to five years of age. We also looked at correlation between SN-5 scoring and asthma control based on TRACK and ACT questionnaires using Pearson correlation analysis. A multivariate analysis was performed comparing SN-5 scores with asthma impairment factors, pulmonary function tests and validated asthma questionnaires. Results were considered significant for *p* value ≤ 0.05. Statistical analysis was performed on all data collected using SPSS v22.0 (SPSS, Chicago, IL, USA). Institutional review board approval (Pro00006181) was obtained prior to collection of data.

## 3. Results

A total of 76 children with asthma were included in this study. Ages ranged from 1 to 19 years, with a mean age of 6.9 years. A total of 38% of the children studied were female. Mean BMI percentile was 69%, with 39% of subjects greater than the 85th percentile. Three-quarters of the children included in this study self-identified as African-American. SN-5 scores did not show significant association with age (*p* = 0.59), gender (*p* = 0.87) or BMI percentile (*p* = 0.17) (Table 1). Reviewing comorbidities associated with asthma, we identified significant association between SN-5 scoring and allergic rhinitis with SN-5 scores greater than or equal to 3.5 having a higher incidence (*p* = 0.01). There were no significant associations with other self-reported conditions often associated with asthma such as eczema and gastroesophageal reflux (Table 2).

Review of pulmonary function testing within this cohort was limited to subjects ≥5 years of age. No single spirometry parameter demonstrated significance when compared to SN-5 score. Children with higher SN-5 scores appeared to have higher fractional exhaled nitric oxide measurements, although this did not reach statistical significance (Table 3).

**Figure 1:**
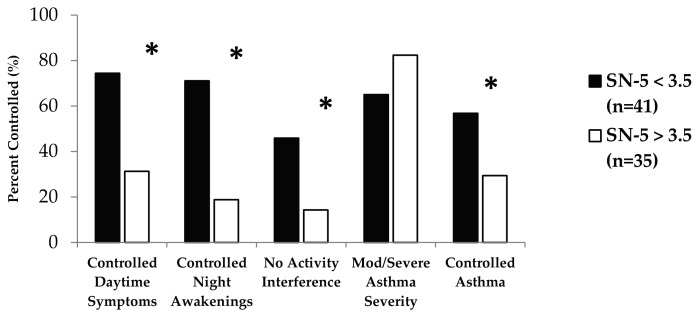
SN-5 score with National Asthma Education and Prevention Program (NAEPP) impairment level and asthma control. Elevated SN-5 score was associated with decreased symptomatic asthma control. * *p*-value ≤ 0.05.

Impact of SN-5 scores on asthma symptoms and impairment was evaluated using NAEPP asthma control criteria. Based on previously published SN-5 scoring cutoffs, we found that elevated SN-5 scoring (≥3.5) was associated with specific levels of NAEPP impairment (Figure 1). This included decreased control of reported daytime symptoms (odds ratio (OR): 0.16; 95% confidence interval (CI): 0.06–0.44), night time awakenings (OR: 0.09; 95% CI: 0.03–0.29), and activity interference (OR: 0.2; 95% CI: 0.06–0.68). These findings are consistent with the finding that SN-5 ≥ 3.5 was associated with decreased asthma control (OR: 0.32; 95% CI: 0.12–0.85) based on NAEPP criteria. While there was no association between SN-5 score and overall asthma severity as defined by the NAEPP, this value did approach statistical significance (*p* = 0.094).

**Figure 2:**
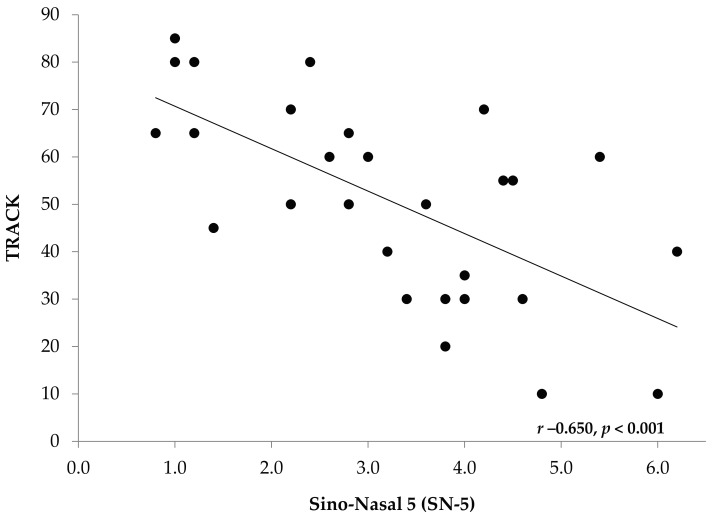
Correlation of Test for Respiratory and Asthma Control in Kids (TRACK) to SN-5 Scores. Inverse correlation between SN-5 score and asthma control using validated TRACK questionnaire for younger children.

**Figure 3:**
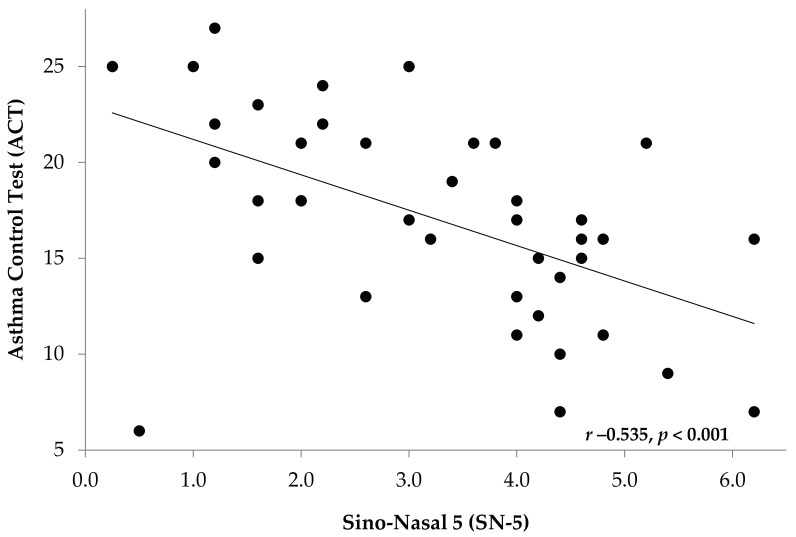
Correlation of Asthma Control Test (ACT) to SN-5 Scores. Inverse correlation in older children between SN-5 score and validated asthma control using ACT questionnaire.

Linear regression analyses revealed a significant inverse correlation between SN-5 score and validated asthma control using validated questionnaires (Figure 2 and Figure 3). This was noted in older children using ACT (*r* −0.535, *p* < 0.001), and in younger children using TRACK (*r* −0.650, *p* < 0.001).

## 4. Discussion

While previous studies have looked at the use of the Sino-Nasal questionnaire in relation to surgical interventions and radiological procedures, this study aimed to identify the impact of upper airway symptoms on control of asthma in children. As a previously validated screening tool, the SN-5 questionnaire is an effective, low-cost and easily acceptable test to identify a disease that is commonly associated with upper airway comorbidities that are not routinely screened. In particular, we recommend primary pediatric providers use this tool in a similar fashion as other routinely-used validated questionnaires for asthma control to help identify upper airway symptoms and conditions. This would allow providers to subsequently initiate and monitor medical management for nasal and sinus symptoms while continuing asthma treatment allowing for effective respiratory control prior to any surgical intervention.

Rhinitis and sinusitis represent significant co-morbidities seen in asthma that manifest as chronic upper airway symptoms. Previous studies have identified allergic rhinitis with a prevalence of 85% in college students with asthma [15]. In children, allergic rhinitis starting in the first year of life was associated with more respiratory symptoms by school age, and an associated increase in asthma diagnosis [16]. Studies in adult asthma patients have also identified 75% of asthma patients as having CRS symptoms [17]. While the epidemiologic link between CRS and asthma has been identified by pathophysiologic and therapeutic observations, these symptoms are often difficult to discern from those originating from the lower airway [18,19]. The presence of CRS has also been associated with more severe, difficult to control asthma [20,21]. While successful CRS management positively affects associated lower airway disease, current literature and clinical practice guidelines lack a subjective tool for assessment of CRS burden for primary care providers, particularly in children [5]. Our study is the first to identify a correlation between previously validated CRS and asthma control questionnaires in a pediatric asthma population.

Overall, we found our population characteristics were similar to previous studies on inner-city pediatric asthma [22,23]. Demographic data suggests an association between asthma with male gender and higher BMI percentiles. This is in keeping with previously-published research [24]. The SN-5 questionnaire was subsequently applied to collected data through a blinded review independent of the physician diagnosing rhinitis. Higher SN-5 scores were associated with allergic rhinitis in keeping with previous validation studies looking at upper airway disease often concomitant in asthma, notably allergic rhinitis. When looking at the rest of the respiratory system, the SN-5 questionnaire did not accurately reflect other clinically-diagnosed comorbidities that were not directly related to the upper airway. It should be noted that not all subjects underwent allergy testing to confirm allergic rhinitis, but rather were defined as having allergic rhinitis based on symptoms and history (i.e., physician-diagnosed), which is an acceptable practice both clinically and in terms of research [15,16,17]. It is interesting that we still identified the association between SN-5 results and clinically-identified allergic rhinitis using this definition. As expected, comorbidities such as eczema, gastroesophageal reflux and sleep disordered breathing were not accurately identified through SN-5 assessment. As an easy-to-use, validated clinical tool, SN-5 is a good means of evaluation for upper airway respiratory disorders concomitant in pediatric asthma.

As a test of primarily lower airway lung dynamics, pulmonary function testing was not seen to have significant association with SN-5 scoring. On a whole, both cohorts involved (significant SN-5 scoring vs. insignificant SN-5 scoring), did have an even distribution of impaired lung function, with a majority across both groups having Forced Expiratory Volume in 1^st^ second (FEV_1_) < 80%, reflecting more severe asthma, but no significant association with SN-5 scoring was seen. Positive SN-5 scoring, ≥3.5, suggests an upper respiratory tract issue with no significant association with pulmonary function or lower airway dynamics. One pulmonary test, fractional exhaled nitric oxide, is used as a quantitative measurement of eosinophil (i.e., allergic) mediated airway inflammation. Our data suggests that children with higher SN-5 scores have association with increased FeNO measurements, although true significance was not seen. There does, however, appear to be a trend towards significance, which might relate to a higher incidence of allergic rhinitis in subjects with elevated FeNO measurements. Ultimately, due to limitations in the population size, future prospective studies with larger study cohorts would need to be conducted to further address the significance of this trend.

When assessing asthma symptoms, current validated scoring systems are in place to evaluate the lower airways, but fail to adequately assess the effects of the upper airway. In our study, asthma impairments seen in daytime-, nighttime- and activity-related symptoms were associated with a significant SN-5 score. We also found significant SN-5 scoring to be associated with decreased asthma control, as seen on TRACK and ACT questionnaires. Both questionnaires are validated tools for assessing disease control in children with asthma [12,13]. This suggests that SN-5 may be an important adjunct tool when assessing asthma severity and control. In asthmatics that lack control despite appropriate medical management, evaluating comorbidities is essential, and the SN-5 questionnaire can draw appropriate attention to CRS and the upper airway. As our results suggest, identification of upper airway symptoms and their contribution to perceived asthma symptoms can be addressed with use of the SN-5 in children with asthma. This may lead to an overall perceived improvement in asthma care and subsequent decrease in the need for asthma therapies.

As a retrospective study, we are limited to identifying associations and are unable to define causality of our findings. An increase in population size will help elucidate any further correlation. In the future, we will undertake a prospective study in a larger cohort using stringent definitions for collected data to further validate the observations of this study. Specifically, we will evaluate whether using SN-5 in an asthma population leads to medical interventions for the upper airway, and subsequent decrease in upper airway and asthma symptoms. With respect to using physician-diagnosed allergic rhinitis to describe the co-morbidity noted to be associated with this questionnaire, in future studies it would be beneficial to include objective testing (skin prick testing, serology) to help define allergic rhinitis, or at least use it as a separate variable when describing the patient population. Additionally, if asthma symptoms are decreased, it would be interesting to see if this could then lead to a decrease in asthma therapies without loss of asthma control.

## 5. Conclusions

This study highlights the value of a screening tool for common comorbid conditions that affect perception of asthma control. Our study is the first to draw attention to the relation of SN-5 scoring with NAEPP guidelines, TRACK and ACT questionnaires. SN-5 can be used as an objective measure to adequately identify upper airway symptoms in the pediatric asthma population, as this does not currently exist. We encourage general pediatric providers to add SN-5 to questionnaires used in daily practice, like ACT and TRACK. Additionally, this tool should be recommended by NAEPP for evaluation of upper airway comorbidity affecting asthma control, and should be used to help initiate and monitor CRS symptoms longitudinally. In pediatric patients with positive SN-5 questionnaires, medical management should be initiated with further referral to otolaryngology and allergy in instances where symptoms persist. From a practical standpoint, SN-5 can be a less expensive indicator to appropriately start rhinosinusitis treatment and minimizing asthma therapies, with referral to subspecialists for those patients in whom SN-5 scoring does not improve on reassessment after initiation of CRS medications. 

## Figures and Tables

**Table 1:** Demographic Data.

Characteristic	Total	SN-5 < 3.5	SN-5 ≥ 3.5	*p* Value
Gender, % Female (*n*)	38.2 (76)	39 (41)	37.1 (35)	0.87
Age, mean years (SEM)	6.9	6.6 (0.6)	7.2 (0.9)	0.59
BMI Percentile, mean (SEM) (*n* = 63)	68.6 (2.9)	63.5 (5.8)	74.1 (4.7)	0.17
BMI ≥ 85%, % (*n* = 63)	39.7	36.4	43.3	0.57

There was no significant difference in demographic data between those with high and low SN-5 scores. BMI: Body Mass Index; SEM: Standard Error of the Mean; SN-5: Sino-Nasal 5 score.

**Table 2:** Co-morbidities Associated with Asthma.

Reported Co-Morbid Conditions	Total (*n* = 76)	SN-5 < 3.5 (*n* = 41)	SN-5 ≥ 3.5 (*n* = 35)	*p* Value
Allergic Rhinitis, %	74.7	62.5	88.6	0.01
Eczema, %	64	65	62.9	0.85
Gastroesophageal reflux, %	21.3	17.5	25.7	0.39
Sleep Disordered Breathing, %	10.7	7.5	14.3	0.34

We identified significant association between SN-5 scoring greater than or equal to 3.5 and allergic rhinitis.

**Table 3:** Pulmonary Function Tests Related to SN-5.

Pulmonary Function Testing	Total (*n* = 39)	SN-5 < 3.5 (*n* = 21)	SN-5 ≥ 3.5 (*n* = 18)	*p* Value
Baseline FEV_1_, Liters (SEM)	1.66	1.59 (0.1)	1.74 (0.2)	0.6
FEV_1_ > 80% predicted, % subjects	48.7	47.6	50	0.9
Baseline FVC, Liters (SEM)	2.0	2.0 (0.2)	2.1 (0.3)	0.7
Baseline FEV_1_/FVC, % (SEM)	83.5	83.3 (1.9)	83.7 (2.5)	0.9
FEV_1_/FVC > 85, % subjects	59	57.1	61.1	0.8
Baseline FEF25–75%, L/min (SEM)	1.7	1.6 (0.2)	1.9 (0.3)	0.4
FeNO, ppb (SEM) (*n* = 15)	36.8	26.4 (7.5)	47.3 (16.3)	0.3

No single spirometry parameter demonstrated significance when compared to SN-5 score. FEF: Forced Expiratory Flow; FEV_1_: Forced Expiratory Volume in 1 second; FVC: Forced Vital Capacity; ppb: parts per billion; SEM: Standard Error of the Mean.
